# Radiotherapy followed by DICEP regimen in treatment of newly diagnosed, stage IE/IIE, extranodal NK/T‐cell lymphoma patients

**DOI:** 10.1002/cam4.3207

**Published:** 2020-06-09

**Authors:** Yizhen Liu, Kai Xue, Zuguang Xia, Jia Jin, Jiachen Wang, Hui Sun, Fangfang Lv, Xiaojian Liu, Junning Cao, Xiaonan Hong, Ye Guo, Xuejun Ma, Qunling Zhang

**Affiliations:** ^1^ Department of Medical Oncology Fudan University Shanghai Cancer Center Shanghai China; ^2^ Department of Oncology Shanghai Medical College, Fudan University Shanghai China; ^3^ Department of Medical Oncology Shanghai East Hospital, Tongji University School of Medicine Shanghai China; ^4^ Department of Radiation Oncology Fudan University Shanghai Cancer Center Shanghai China

**Keywords:** DICE, NK/T‐cell lymphoma, Pegaspargase, radiation

## Abstract

**Background:**

The optimal treatment strategies for extranodal natural killer/ T‐cell lymphoma (ENKTL) have not been defined. We conducted this prospective, open‐label, phase II, single‐center study aimed to explore the efficacy and safety of radiotherapy followed by DICEP (Dexamethasone, ifosfamide, cisplatin, etoposide, and pegaspargase) regimen in the treatment of patients with untreated, stage IE/IIE, extranodal NK/T‐cell lymphoma.

**Methods:**

Thirty eligible patients were enrolled in this study, receiving radiotherapy of 50Gy/25fx, and followed by chemotherapy with DICEP regimen for 3 cycles if tolerated. Median follow‐up time of this study was 70.8 months. We constructed Kaplan‐Meier survival curves for survival analyses.

**Results:**

The most common manifestations at the onset of disease were nasal obstruction (80%), with or without fever, and pharyngalgia (20%). The overall response rate (ORR) was 96.7% (29/30). Four patients (13.3%) had progression of the disease (PD), the estimated 5‐year progression‐free survival (PFS) rate was 86%. Four patients (13.3%) died of disease, and the estimated 5‐year cumulative overall survival (OS) was 87%. The most common hematological toxicity was grade 3 or grade 4 neutropenia, which could be successfully managed via using growth‐stimulating factors or dose modifications. Hypoalbuminemia and decreased fibrinogen are the top two nonhematologic toxicities. No treatment‐related death occurred in this study.

**Conclusions:**

Our present study showed that radiotherapy followed by DICEP chemotherapy could be an effective and tolerable treatment modality for newly diagnosed, stage IE/IIE ENKTL patients. Adverse events were predictable and manageable.

**Trial registration:**

ClinicalTrials.gov Identifier: NCT01667302.

Registered: 1 July 2012;

## Graphical abstract

The current results indicated that radiotherapy followed by DICEP chemotherapy could be an effective and feasible treatment strategy for patients with stage IE/IIE ENKTL.

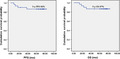

## 1 BACKGROUND

Extranodal NK/T‐cell lymphoma (ENKTL) accounts for approximately 12% of peripheral T‐cell lymphoma, and constitutes 5%‐10% of non‐Hodgkin lymphoma (NHL) cases in East Asia.^1^, ^2^ Most ENKTL originates from nasal and paranasal areas.^3^ More than 70% patients were presented with stage I or stage II disease at diagnosis.^4^

At present, the optimal treatment strategy for this disease has not been fully identified. In previous reports, the combination of extensive or modified involved‐field radiotherapy with chemotherapy can improve the treatment outcome of early‐stage patients compared with radiotherapy alone.^5^ Because of the overexpression of the multidrug‐resistant (MDR1) gene, conventional chemotherapies such as anthracycline‐based regimen, CHOP, or CHOP‐like regimens could not provide promising results.^6^, ^7^, ^8^ Cisplatin and ifosfamide are not the substrate of P glycoprotein and may be a good option for the treatment of ENKTL. The development of nonanthracycline contained regimen which incorporates etoposide, ifosfamide, and platinum also has improved outcomes of ENKTL.

L‐asparaginase exhibited a specific anticancer mechanism which is not influenced by P glycoprotein, making L‐asparaginase‐based chemotherapy highlighted recently as an effective treatment for ENKTL.^9^, ^10^, ^11^ However, it has the drawback of short plasma half‐life and allergic reactions, which limit its use in clinical application. Pegaspargase is the PEGylated form of L‐asparaginase, which could reduce the host immune system activation, decrease degradation rate of the drug by plasmatic proteases, and has a comparable cost.^12^, ^13^

Here, we designed a prospective, single‐center, phase II clinical trial, investigating the efficacy and tolerance of radiotherapy followed by DICEP (dexamisone, ifosfamide, cisplatin, etoposide, and pegaspargase) regimen in the treatment of newly diagnosed, stage IE/IIE, extranodal NK/T‐cell lymphoma (*Trial registration: ClinicalTrials.gov* NCT01667302; *Registered:* 1 July 2012). This study was approved by the Institutional Review Board of the Fudan University Shanghai Cancer Center.

## 2 PATIENTS AND METHODS

### 2.1 Patients

The inclusion criteria for this study were as follows: (a) 18‐70 years old; (b) histological confirmed, newly diagnosed stage IE and IIE extranodal NK/T‐cell lymphoma; (c) Eastern Cooperative Oncology Group (ECOG) status of 0‐1; (d) adequate hematologic, hepatic, and renal functions; (e) life expectancy > 3 months; (f) at least one measurable target lesion. Exclusion criteria included: (a) low risk population (stage I without B symptoms, local invasion, and elevated LDH level); (b) lactating or pregnant women; (c) prior history of pancreatitis; (d) had contraindication of steroid, such as uncontrolled diabetes; (e) serious infection and uncontrolled diseases; and (f) history of malignancies, other than cured skin basal cell carcinoma and carcinoma in situ of uterine cervix. All patients in our study signed informed consent forms.

### 2.2 Treatment

After diagnosis and staging, patients in our study were firstly treated with intensity‐modulated radiation therapy (IMRT), and then were referred for chemotherapy with DICEP regimen (D1‐4: dexamethasone 40 mg, ifosfamide 1200 mg/m^2^, cisplatin 20 mg/m^2^, etoposide 60 mg/m^2^; D1: pegaspargase 2000 U/m^2^, max 3750IU) for 3 cycles if tolerated. Radiotherapy was delivered with IMRT techniques using 6MV photons. All patients received computed tomography simulation scanning of the head and neck region with thermoplastic mask for the treatment volume delineation and treatment planning before IMRT. The extensive involved field was defined as previously described.^14^ The total dose of upfront radiotherapy was 50 Gy in 25 fractions, 5 days per week.^15^ Chemotherapy began at 4 weeks after the completion of radiotherapy and no more than 6 weeks. DICEP was administered every 3 weeks. Granulocyte colony‐stimulating factor (G‐CSF) could be administered by subcutaneous injection during myelosuppression. Prophylactic usage of G‐CSF is recommended in patients more than 60 years old and those who suffered from grade 4 neutropenia or grade 3 neutrophil with fever in the previous cycle of treatment.

All adverse events during treatment were recorded according to version 4.0 of National Cancer Institute Common Terminology Criteria of Adverse Events. During chemotherapy intervals, blood routine test, hepatic, and renal functions should be monitored. If patients had grade 4 hematologic toxicity or grade 3 neutrophil with fever, then the next cycle doses of all chemotherapy drugs were reduced by 25% except pegaspargase. If grade 4 hematologic toxicity appeared once again, dose of pegaspargase was reduced by 25%. Before each cycle, patients should have neutrophil count more than 1.5 × 10^9^/L, platelet count more than 75 × 10^9^/L to receive the chemotherapy regimens. And all other adverse events (AEs) should return to grade 1.

### 2.3 Response evaluation

All patients received whole‐body PET‐CT scan, enhanced CT, and/ or MRI scan of the involved area at baseline staging before treatment. Response evaluation was performed by CT and/or MRI at the end of radiotherapy. PET‐CT was applied in 4‐6 weeks after completion of chemotherapy of DICEP regimen. Treatment response was recorded based on the Cheson 2007 criteria. After the completion of therapy, follow‐ups were carried every 3 months for the first 2 years, and every 6 months for the next 3 years, and annually thereafter. The follow‐up visits included a physical examination, routine blood tests, selective endoscope, and CT/ MRI scan of the involved area for each patient.

### 2.4 Statistical analysis

All analyses were carried out using PASW statistics 18. Overall survival (OS) was calculated from the date of diagnosis to the date of death or the date of last follow‐up. Progression‐free survival (PFS) was measured from the date of diagnosis to the date of identification of disease progression. We constructed Kaplan‐Meier curves for survival analyses.

## 3 RESULTS

### 3.1 Patient characteristics

A total of 30 patients (22 males, 8 females) were enrolled in this study from June 2012 to July 2014. At the time of diagnosis, the median age was 45 years old (range: 26‐61 years). The most frequent clinical characteristics at the onset of disease were nasal obstruction (80%) with or without fever and pharyngalgia (20%). Nine (30%) patients were at stage IE, 21 (70%) were at stage IIE based on Ann Arbor staging criteria. KPI score (including stage III/ IV, presence of B symptoms, lymph node involvement, and high LDH level) was available in all the patients. Accordingly, 20 patients were KPI 0 or 1 and 10 patients were KPI 2 or more. B symptoms present in 17 (56.7%) patients at the diagnosis of the disease. The clinical and laboratory parameters of the patients are shown in Table 1.

**TABLE 1:** Baseline Characteristics of 30 patients with nasal, extranodal NK/T‐cell lymphoma

Characteristic	No. of patients	%
Gender		
Male	22	73.3
Female	8	26.7
Age at diagnosis		
>60	1	3.3
≤60	29	96.7
Ann Arbor stage		
IE	9	30.0
IIE	21	70.0
LDH level at diagnosis		
Normal	27	90.0
Elevated	3	10.0
NKPI		
0‐1	20	66.7
≥2	10	33.3
Fever at diagnosis		
Yes	16	53.3
No	14	46.7
B symptoms		
Yes	17	56.7
No	13	43.3
Ki67 (n = 26)		
<80%	15	57.7
≥80%	11	42.3
Lymoph node involvement		
Yes	14	46.7
No	16	53.3

### 3.2 Treatment and response

**FIGURE 1:**
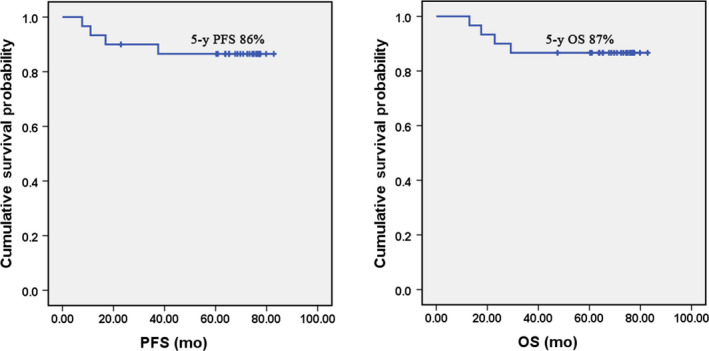
PFS and OS of NK/T patients in our study The PFS (left) and OS (right) of stage IE/IIE NK/T patients in our study

After radiotherapy, 16 patients achieved complete response (CR) and 14 patients got partial response (PR). Twenty‐nine (96.7%) patients completed the entire course of treatment, one patient received radiotherapy followed by only 1 course of DICEP due to severe pneumonia. At the end of chemotherapy, 96.7% (29/30) achieved CR. With a median follow‐up of 70.8 (13‐82.8) months, 4 disease progression events were observed. Local recurrence within radiation field was observed in 1 patient 11 months after treatment, whereas distant relapse occurred in 3 patients at a median of 16.9 months (7.7‐37.5 months). The estimated 5‐year PFS rate was 86%. Four patients died of disease. The estimated 5‐year OS rate was 87% (Figure 1).

Patients showed no grade 3/4 hematologic toxicities during radiation. Four patients experienced grade 3 mucositis. Other mild adverse effects such as dry nose and dysphagia were well tolerated. The adverse events during DICEP therapy are exhibited in Table 2. Hematological toxicity was the predominant adverse event. Grade 3/4 neutropenia occurred in 96.5% patients and febrile neutropenia in 16.7% (5/30) patients, which led to dose reduction of DICE in 70% (21/30) patients since second course. In our study, 11 patients received prophylactic usage of G‐CSF.

**TABLE 2:** Adverse event from chemotherapy and event grade

Toxicity	No. of events			No. of events		
	Grade 1	Grade 2	% Grade1 + 2	Grade 3	Grade 4	%Grade 3 + 4,
Hematologic						
Neutropenia	0	1	3.3	6	23	96.7
Anemia	2	12	46.7	9	1	40.0
Thrombocytopenia	1	6	23.3	6	2	26.7
Nonhematologic						
Nausea	3	5	26.7	2	0	6.7
Increased ALT/AST	11	0	36.7	0	0	0
Hyperbilirubinemia	6	4	40.0	1	0	3.3
Hypoalbuminemia	7	20	90.0	0	0	0
Decreased fibrinogen	4	9	43.3	5	1	20.0

Hypoalbuminemia and decreased fibrinogen were the two common nonhematologic toxicities. All hypoalbuminemia in our study was grade 1 and 2. Grade 3 and grade 4 decreased fibrinogen occurred in 20% (6/30) patients. Pegaspargase was obviated in 3 patients from second course and delivered with decreased dose by 25% since third course in one patient. No treatment‐related death was observed in our study.

## 4 DISCUSSION

Extranodal NK‐T‐cell lymphoma (ENKTL) has a racial and geographical predisposition. It is more common in Asia than in Western countries. Optimal treatment remains unclear. Radiotherapy plays a significant role in localized ENKTL. Nevertheless, owing to the relative high treatment failure, mainly distant and locoregional relapse, combined modality with radiotherapy and chemotherapy become the widely accepted approach for localized ENKTL with risk factors.^16^ For patients with stage I/II ENKTL, the sequence of radiotherapy and chemotherapy is debatable. It could be concurrent chemoradiation, sequential chemotherapy after radiotherapy, or chemotherapy with sandwiched radiotherapy.^17^, ^18^, ^19^ Some studies illustrated the superior treatment results of upfront radiotherapy.^20^ Hence, we designed this phase II study to evaluate the treatment efficacy and tolerance of radiotherapy followed by chemotherapy with DICE regimen as well as peg‐asparaginase which was found to be effective in ENKTL. Previous study showed that the radiation dose of < 45Gy was significantly associated with local relapse. Radiotherapy dose equal to or more than 50 Gy brings on better response than dose less than 50 Gy, whether concurrent or sequential chemoradiation.^21^ We employed radiation with 50Gy in our study and showed promising results.

Our results indicate that sequential radiotherapy followed by DICEP therapy is effective in treating early‐stage ENKTL. The CR rate was 96.7%. Five‐year PFS and OS rates were 86% and 87%, respectively. The results in our study are better than previous reports of radiotherapy alone or in combination of other chemotherapy regimens in the treatment of the disease.^22^, ^23^ Previous studies showed that combined radiotherapy with chemotherapy containing L‐asparaginase/ pegaspargase regimens exhibited 5‐year OS of 60%‐85% and 5‐year PFS of 64%‐74%.^24^, ^25^, ^26^ It was reported that SMILE followed by radiotherapy had high efficacy in previous study.^27^ However, the main deterrent for the use of SMILE is its toxicity. This regimen associated with severe myelosuppression and infection, even treatment‐related death.^28^ A recent study reported that stage I/II newly diagnosed ENKTL patients treated with EPOCHL (EPOCH + L‐asparaginase) regimen combined with radiotherapy had the 5‐year OS and PFS of 65.3% and 59.8%, respectively.^29^ Another study published in 2020 evaluating the clinical efficacy of LVD (L‐asparaginase, vincristine, and dexamethasone) combined with intensity‐modulated radiation therapy (IMRT) in early‐stage ENKTL patients showed the 5‐year OS and PFS of 74.3% and 73.1%.^30^ However, both were retrospective studies.

Also there were studies showing promising 3‐year PFS and 3‐year OS data.^31^, ^32^ However, the long‐term efficacy of these treatment modalities is still not known.

Concerning safety, the most common adverse event resulting in dose reduction was neutropenia, accounting for 70% in our study. It could be successfully addressed by using growth factors or dose modifications. Other common AEs were grade 1 or grade 2 hypoalbuminemia, anemia, and hyperbilirubinemias. All the toxicities but pneumonia were tolerable without interruption of treatment. Moreover, during the 5‐year follow‐ups, we did not observe long‐term chemotherapy‐related toxicity among survivors.

We noticed that the percentage of grade 3 or 4 neutropenia occurred commonly in our study, and led to dose reduction of DICE in 70% patients since second course. However, the outcome of treatment achieved a high ORR and relatively high 5‐year PFS and OS. Besides, our study exhibited the long‐term follow‐up data and suggested DICEP regimen may be a promising treatment in ENKTL.

These better results in our study may be due to the proper sequence of radiotherapy and chemotherapy as well as the appropriate radiotherapy dose and chemotherapy regimen we applied. Close monitoring during treatment and follow‐up were also contributing to the promising results. However, we could not deny the fact that patients selection may had an impact in the favorable disease outcome, as most patients were under 60 years old and with relative good performance status in our study. We also could not exclude the limitation of relative small number of patients, as the incidence of ENKTL is low, which limit us to enroll a large number of patients. Our results may shed light on novel ways in the treatment of newly diagnosed, stage IE/IIE, ENKTL patients and inspired us to conduct further multicenter studies to develop optimal management strategies of this disease.

## 5 CONCLUSIONS

Our results indicated that sequential radiotherapy followed by DICEP chemotherapy is effective and feasible for the treatment of stage IE or IIE, ENKTL. All adverse events were predictable and manageable. The initial decreased dosage of DICEP or combined with long‐acting G‐CSF prophylaxis may improve safety in the further clinical practice without the loss of efficacy.

## COMPETING INTERESTS

Nothing to disclose.

## AUTHOR CONTRIBUTIONS

YZL analyzed and interpreted the patients' data, and have drafted the work. KX, ZGX, and JJ have made substantial contributions to acquisition data. JCW and HS performed the follow‐up work. FFL, XJL, JNC, XNH, and YG designed and supervised the work. XJM and QLZ substantively revised the manuscript.

## Data Availability

The data that support the findings of this study are available from the corresponding author upon reasonable request.
